# Prevalence and Associated Factors of Malaria Infection among Outpatients Visiting Shewa Robit Health Center, Northcentral Ethiopia

**DOI:** 10.1155/2022/1784012

**Published:** 2022-03-23

**Authors:** Azene Tesfaye, Tadegew Teshome

**Affiliations:** ^1^Department of Medical Laboratory Science, College of Medicine and Health Sciences, Arba Minch University, Arba Minch, Ethiopia; ^2^Department of Zoological Sciences, College of Natural Sciences, Addis Ababa University, Addis Ababa, Ethiopia; ^3^Shewa Robit Secondary and Preparatory School, Ministry of Education, Addis Ababa, Ethiopia

## Abstract

**Introduction:**

Malaria infection is a serious health problem killing millions in tropical developing countries including Ethiopia. The present study focused on assessing malaria prevalence and identification of determinants in Shewa Robit, northcentral Ethiopia.

**Methods:**

A cross-sectional study was conducted among 422 participants who visited Shewa Robit Health Center between 01/10/2017 and 30/04/2018, using a simple random sampling. Sociodemographic characteristics were recorded using a pre-tested semi-structured questionnaire and infection was confirmed by microscopic examination. Data were analyzed using the Statistical Program for Social Sciences (SPSS) version 20 and *p* < 0.05 was used to indicate the level of significance.

**Results:**

Eighty-one (19.0%) microscopically confirmed malaria cases were recorded, *P.vivax* was the most frequently detected species (*n* = 58; 71.6%). Interestingly, 73.2% (*n* = 309) of the participant did not utilize LLINs due to the fear of toxicity (37.4%, *n* = 158), misconception (21.6%, *n* = 91), and shortage (14.2%, *n* = 60). The data showed age, gender, marital status, family size, usage of LLINs and application of IRS, proximity to mosquito breeding sites and less robust and porous walls were the determinants of the infection in the study area.

**Conclusion:**

The prevalence of malaria in the study population was high and *P. vivax* being the most common causative agent. Environmental and behavioral factors related to LLIN are the potential determinants of malaria. Continued public health interventions, targeting proper utilization of bed nets, drainage of stagnant water, and improved public awareness about reducing the risk of insect bites have the potential to minimize the infection.

## 1. Introduction

Malaria is recognized around the world as a debilitating and terrible infectious disease that kills millions and causes serious complications such as severe anemia, cerebral involvement, acute renal failure, and hypoglycemia [1, 2]. It is distributed worldwide including Africa, south and Central America, south and southeast Asia, particularly with very high transmission intensity in the Sub-Saharan Africa [3]. A recent WHO report on global malaria status revealed an estimated 241 million cases and 627,000 deaths in 2020 [2].

Malaria is widespread in almost 45 African nations, including Ethiopia, with nearly 3 million cases each year, and morbidity and mortality rates are growing substantially [4]. It is distributed almost everywhere in the country and affects about 70% of the population [5]. Previous research in Ethiopia has shown that a large part of the geographic and agroecological environment (68%) is conducive to the transmission of malaria, with altitude and rainfall appearing extremely significant. In particular, 75% of the topography is below 2000 meters from sea level, supporting its transmission [6, 7]. Research report confirmed that around 68% of the total population live in malaria-prone areas with more than 50 million people are at risk of malaria, with an estimated 4-5 million cases and 70,000 deaths each year [8, 9]. In addition, transmission follows the rainy season and occurs between September and December in almost every part of the country, while a minor transmission season occurs between April and May [10].

Previous research confirmed that age, sex, marital status of the respondent [5], proximity to mosquito breeding sites such as stagnant water [7], temperature, humidity, precipitation, education, occupation, and income are the main risk factors that favor the transmission of malaria [11, 12].

As part of the global Roll Back Malaria project, Ethiopia has a long-term goal to eliminate malaria [13], and in this context, the government adopts a variety of interventional measures. Early detection and treatment, selective vector control measures such as indoor residual spray (IRS) and long-lasting insecticidal nets, (LLINs), and environmental management are important steps. In addition, quick diagnostic tests are performed along with the adaptation of artemisinin-based combination therapy [5, 14].

Malaria remains one of the most serious public health concerns in Ethiopia, despite significant efforts to combat it [15]. Meanwhile, the disease is much more prevalent in rural areas due to favorable conditions for the establishment and proliferation of associated vector [16]. However, studies have already shown that malaria transmission has increased in metropolitan settings [17, 18]. This could be linked to the growing urbanization, with a lack of proper sanitation, substandard housing, and poor surface water drainage, all of which enhance the exposure to mosquitoes and subsequent disease transmission [16–18]. Furthermore, poor health services, increased migration of people from malaria affected rural area to urban sites, limited extent of indoor residual insecticide spraying (IRS) and bed net use, an increase in the number of man-made mosquito breeding sites, unplanned irrigation schemes and water reservoirs may hasten the spread of the disease to urban habitats [17].

The terrain of Shewa Robit is favorable for the transmission of malaria, which is one of the top ten causes of morbidity (Health Office report, 2018). Despite the high rate of malaria infection, literature analyzes showed a paucity of recently updated data on the prevalence and risk factors in the study area [7]. In fact, effective public health programs to control and prevent malaria require current and consistent data on prevalence and existing risk factors [2]. However, the prevalence of malaria in the research area has been poorly studied. Therefore, the aim of this study was to determine the prevalence of malaria infection and its associated factors among suspected outpatients visiting Shewa Robit Health Center, northcenteral Ethiopia.

## 2. Materials and Methods

### 2.1. Study Area, Design and Study Population

A cross-sectional study was conducted among all malaria suspected outpatients who visited Shewa Robit Health Center located in Shewa Robit town, from 01/10/2017 to 30/04/2018. The town is located 225 km northeast of Addis Ababa, in the Amhara Regional State at an elevation of about 1,280 meters above sea level. The town lies at a longitude and latitude of 10°060 N39°590 E and 10.1°N39.983°E, respectively. The climate is tropical with an annual temperature ranging from 28 to 37°C with an annual rainfall of 1000 mm. It has nine administrative units (Kebele) with a total population of 50,528, of which 25,890 (51.2%) are women. Malaria is one of the top ten diseases in the town and is reported throughout the year [14], and the highest transmission rate usually occurs twice a year, from September to November and from June to August. The town is classified as a malarious area, with the disease spreading to the level of an epidemic once in every five years [1].

All suspected cases with a febrile illness (>37.5 C) who have been living in the administrative units of Shewa Robit for at least six months were included in the study. However, those who underwent chemotherapy with antimalarial drugs three months prior to the start of the study were excluded.

### 2.2. Sample Size Determination and Sampling Methods

The sample size was calculated using a single population formula. A prevalence of 50% was chosen, as there were no specific reports in the study area (health center). In order to calculate sample size, the value of Z chosen was 1.96 at 95% CI and a 5% margin of error. Therefore, the final sample size was consolidated to 422, after adding 10% non-response rate.

### 2.3. Sampling Procedures and Techniques

To start with, all suspected outpatients i.e, these suspected cases with febrile illness attended to the health center were stratified according to sex and age, and a random sampling technique was used to select each participant from the kebeles chosen.

### 2.4. Specimen Collection and Processing

After a briefing on the purpose of the study, all participants submitted their informed consents and assents before the commencement of data and sample collection. Thin and thick blood smears have been used with finger pricks to detect the *Plasmodium* infection. Thin films were fixed with 100% methanol, and both thin and thick films were stained with 3% Giemsa according to the protocol [19]. Thick films were then examined under high magnification (100x) for the presence of *Plasmodium* parasites. In the case of the thin films being found to be positive, an investigation for species identification was done. A second expert laboratory technologist who was unaware of the diagnosis by the first reader reexamined all positive slides, as well as a random sample of 10% of negative slides. No disparity has been found between the opinions of the readers.

### 2.5. Data Collection Methods

Sociodemographic situation of the participants (sex, age, kebele, family size, marital status, occupation, income, and educational level), infection related factors (history of infection, availability and use of LLINs, application of IRS, proximity to mosquito breeding site, and holes *b*/*n* wall and roofs) were recorded. A face-to-face interview with well-trained health experts was conducted to obtain the information from each participant. Structured and pre-tested questions in English were prepared and translated into the regional language (Amharic) to ensure the quality and consistency of the data.

### 2.6. Quality Control

Standard operating procedures (in-house SOP manual) were followed during blood collection (aseptic method), preparation of blood smear, staining, and examination of blood films to maintain quality. An experienced laboratory technologist evaluated the quality of the laboratory reagents and instruments. The collection technique was ensured, as was the quality of the samples, and the serial numbers were checked.

### 2.7. Data Analysis

Before being entered into Epi Info 3.5.3 and exported to statistical package for Social Science (SPSS) 20, the data was cleaned, updated, and double-checked (IBM, USA). Frequency and percentage were used to describe the characteristics of the patients. The Pearson chi-square test was performed to examine the association among sociodemographic and topographic characteristics. Variables with a *p* value less than 0.25 were selected as candidates for the multivariable analysis and fitted into a logistic regression model in the bivariable analysis. A statistically significant association was confirmed at a *p* value of <0.05.

### 2.8. Ethical Considerations

The research was ethically approved by the Institutional Review Board (IRB) of Addis Ababa University, and an ethics clearance was provided to the Shewa Robit Health Center [No, S/R/H/C/44/017]. Participants were informed of the minor risks involved in this study, which was also conducted in accordance with the Declaration of Helsinki [20].

## 3. Results

### 3.1. Sociodemographic Characteristics

Data showed that 422 individuals with a mean age of 12.53 ± 0.58 participated and majority (43.6%, *n* = 184) of them were under 5 years. Furthermore, 57.1% (*n* = 241) were unmarried with a family size of more than five members. Majority (36.7%, *n* = 155) of the respondents were an attendants of secondary education and above whereas, 33.9% (*n* = 143) of the participant were illiterate. Most of the study participants were farmers and merchants ie, 23.2% (*n* = 98) and 16.1% (*n* = 68) respectively by occupation with monthly family income of less than 18.30 USD (Table 1).

### 3.2. Seasonal Pattern of Malaria Infection

Although the *Plasmodium* species and extent of malaria infection varied in the study area, it occurred practically in every month and season. The data showed that the highest rate of infection was recorded in October and November with an infection rate of 34.3 (*n* = 23) and 35.1% (*n* = 20), respectively. However, a lower incidence of infection was observed in January with an infection rate of only 4% (*n* = 2). In particular, the findings revealed that *P. falciparum* infection peaked in October and November with an infection rate of 43.5 (*n* = 10) and 20% (*n* = 4), respectively. The lowest infection with *P. falciparum* was recorded in January, February, and April with nil incidence. However, the infection rate caused by the malaria infection by *P. vivax* went up to the maximum in January, February and April (100%, *n* = 16), while the lower infection caused by *P. vivax* was recorded in March with a transmission rate of 60% (*n* = 3). Similarly, mixed infection was recorded in October and November with a prevalence of 21.7% (*n* = 5) and 5% (*n* = 1) respectively (Table 2).

### 3.3. Factors Related to Infection

It was found that 289 (75.3%) of the participants had a history of malaria infection in their households. Although (83.6%) of the participant have access to long-lasting insecticide nets (LLINs), they sleep under the net daily (26.8%) and during the high transmission season (43.1%). However, most of the study participants (73.1%) did not use LLINs for two reasons, fear of toxicity (37.45%) and misconception (21.6%) due to the belief that the net did not prevent infection (Table 3).

### 3.4. Prevalence of Infection

**Figure 1:**
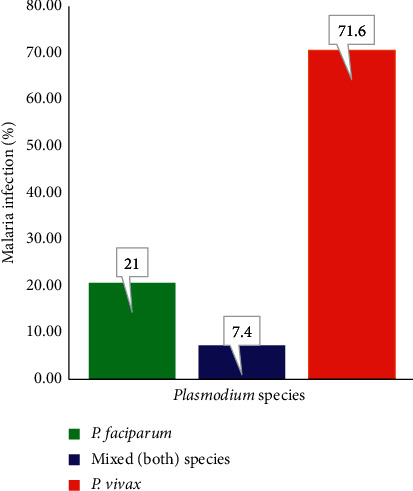
Distribution of *plasmodium* species in Shewa Robit, Ethiopia, 2018.

The findings show that 19% (*n* = 81) of the participants had malaria parasites in their blood that could be seen microscopically. The most prevalent *Plasmodium* species found in individuals with positive laboratory test results were, *P. vivax* 71.6% (*n* = 58), *P. falciparum* 21% (*n* = 17) and mixed infection (recurrence of both species) accounts 7.4% (*n* = 6), as shown in Figure 1.

### 3.5. Factors Associated with Malaria

The bivariable and multivariable analyzes revealed that several factors in the research area contribute to malaria infection. Age, marital status, family size, LLIN use, IRS, proximity to mosquito breeding locations, and the presence of holes in wall and roof are all associated factors. Results suggest that malaria infection was significantly associated with marital status and the family size. Those who were married or having a family size of ≥5 were 4.97 (CI 95%: 2.67–9.28) and 2.20 (CI 95%: 1.2–4.06) more likely to be infected with malaria respectively. The result confirmed that usage of LLTN reduces malaria infection. Study participants who did not use LLTN (CI 95%: 0.69–2.83) were 1.4 more likely to be infected with malaria as compared to their counterparts. Furthermore, study participants who refused IRS (CI 95%: 1.21, 5.60) were 2.6 times more likely than their peers to develop malaria infection. The presence of a mosquito-nesting site close to the house and holes between the house wall and the roof had a strong relationship with the occurrence of malaria infection. According to the findings, study participants who had proximity to mosquito location were 3.91 times more likely to contract malaria than their peers CI 95%: 1.87, 5.18). However, the chance of malaria infection was 2.1 higher in the participant who lived in a house with holes between the wall and the roof (CI 95%: 1.13–3.67) as shown in Table 4.

## 4. Discussion

This study evaluated the prevalence of malaria infection in Shewarobait, Ethiopia from October 2017 to April 2018. The result showed that malaria is still one of the most serious public burdens in the study area. In addition, it was evident that age of the participants, sex, marital status, family size, utilization of LLINs and IRS, proximity to mosquito breeding site, and presence of holes on the wall were determinants of malaria transmission. In the current study, the overall percentage of malaria cases detected was 81 (19%) (*n* = 422), with *P. vivax* being the most prevalent species, is lower than the previous findings from Wolaita Zone (33.27%) [21], and Hallaba (82.84%) [22], However, is higher than that reported Sudan (9.1%) [23] Kenya (18.0%) [24], Kenya (6.4%), Tanzania (12.1%), and Uganda (6.3%) [25]. Interestingly, some earlier studies conducted in Ethiopia showed much lower prevalence. For example, 11.45 and 5.4% corresponding to localities Aresi Negelle [22], and Wortea [26] respectively. These inconsistencies may be due to differences in geographic location and the seasonality of infection. The result showed a male preponderance with an infection rate of 25.9% and it was only 11.9% in case of female. These findings are in line with the outcomes of similar studies conducted in Oromia [5], Kombolcha [27], and Kenya [24]. However, is inconsistent with the results of research done in Woreta [28] and Wolaita Zone which confirmed that females was 1.3 times more likely to be infected [21].

The predominant *Plasmodium* species detected among the participants in the current study participants was *P. vivax.* This is in agreement with previous report from Jimma Town [13], Aresi Negelle [29], Hallaba [22], there exists a the dominance of *P. vivax* over *P. falciparum* in recent years [30]. This could be due to the recurring nature and drug resistance of *P. vivax* against chloroquine [31].

According to the present study, 72.3% of participants had a history of malaria infection however, only 63 (20.7%) were infected with malaria. In particular, individuals who had a family history of malaria were 1.53 times more likely to be infected by *Plasmodium* species compared to their counterparts (*p* < 0.001). These findings were supported by the Hamusite report, northwest Ethiopia [32]. This might be duto to family members with has a history of malaria infection may become reservoirs of *Plasmodium* parasites.

Different sociodemographic and other factors had been analyzed by taking into consideration of prevalence of malaria infection. Of these factors, the age was one of the significantly associated factors. Here, the odds of having malaria infection were 2.31 and 4.05 more likely among participants in the age group 5–14 years and above 15 compared to others. This aspect of the study is comparable to a previous work conducted in Woreta [28], Kombolcha [27], Dembia district [33] and Kola Diba [31] which reported that the prevalence of malaria high in the age group >15 years. This could be related to their frequent outdoor activities, such as agricultural practices related to irrigation during the peak period of malaria transmission [7].

The odds of being infected with malaria were 1.4 and 2.6 times higher among participants who did not use LLIN and apply IRS, respectively, and this is consistent with the results of other studies conducted in Jimma [17] and Shewa Robit [7] which proved that the use of LLIN and IRS and reduced the transmission. Our results showed that living near to mosquito breeding sites increased the probability of being infected. The study also highlighted that participants who lived near mosquito breeding sites was 1.4 more likely to be infected with malaria compared to their counterparts, who resided away. These findings were consistent with the results of an earlier research report from Arba Minch [34] and Jimma [17]. Less and porous walls and roof of household are significantly associated with malaria infection. The study indicates that participants living with such houses were 2.1 times more likely to be infected with malaria, and this is in line with the results of an earlier research done in Shewa Robit research report done in Shewa Robit [15].

## 5. Limitation of the Study

This is a cross-sectional study that addresses percentage prevalence and cannot account for seasonal transmission trends. In addition, the study is based on a single institution and has a shorter duration, including a smaller sample size. All surveys are self-reported without confirmation of bednet ownership and usage, and frequently application of IRS and RDTs. In addition, no PCR tests were performed to identify the infection and the *Plasmodium* species.

## 6. Conclusions

The study population who attended the Shewa Robit Health Center had a high incidence of malaria, with *P. vivax* being the most common causative agent. The main infection factors linked to the infection in the study area were age, sex, marital status, family size, use of LLIN and IRS, presence of mosquito breeding sites, and openings on their wall/roof. In addition, the main reason for rejecting LLIN is misconceptions about the toxicity of the treated net. The burden of malaria could be reduced by focusing on changing the attitudes towards malaria prevention and control through continued health education.

## Figures and Tables

**Table 1:** Sociodemographic characteristics of the respondents in Shewa Robit, Ethiopia (*n* = 422).

Variables		Frequency (*n*)	Percent (%)
Age	<5	184	43.6
	5–14	139	32.9
	>15	99	23.5

Sex	Male	212	50.2
	Female	210	49.8

Marital status	Married	181	42.9
	Unmarried	241	57.1

Faimly size	<5	202	47.9
	≥5	220	52.1

Education level	Illiterate	143	33.9
	Primary and junior school	124	29.4
	Secondary and above	155	36.7

Occupation	Farmer	98	23.2
	NGO worker	44	10.4
	Private business	73	17.3
	Merchant	68	16.1
	Government employee	72	17.1
	Daily labourer	33	7.8
	Student	34	8.1

Monthly income ($)	<18.30	172	40.8
	18.30–78.44	124	29.4
	>78.44	126	29.9

$ United States dollar (USD).

**Table 2:** Seasonal patterns and prevalence of *plasmodium* species in Shewa Robit, Ethiopia (*n* = 422).

Month	Total examined	Total confirmed *N* (%)	*Plasmodium* species			*X* ^2^	*p* value
			*P. falciparum N* (%)	*P. vivax N* (%)	Mixed *N* (%)		
October	67	23 (34.3)	10 (43.5)	17 (73.9)	5 (21.7)	38.89	*p* < 0.001
November	57	20 (35.1)	4 (20.0)	15 (75.0)	1 (5.0)		
December	33	8 (24.2)	1 (12.5)	7 (87.5)	0 (0.0)		
January	50	2 (4.0)	0 (0.0)	2 (100)	0 (0.0)		
February	76	10 (13.2)	0 (0.0)	10 (100)	0 (0.0)		
March	71	5 (7.0)	2 (40)	3 (60)	0 (0.0)		
April	68	4 (4.4)	0 (0.0)	4 (100)	0 (0.0)		
Total	422	81 (19.0)	17 (21)	58 (71.6)	6 (7.4)		

**Table 3:** Factors that contribute to the transmission of malaria infection in Shewa Robit, Ethiopia (*n* = 422).

Variables		Frequency	Percent
History of malaria infection	Yes	305	72.3
	No	117	27.7

Availability of LLINs	Yes	353	83.6
	No	69	16.4

Reason for not using LLINs	Shortage	60	14.2
	Afraid of toxicity	158	37.4
	Misconception	91	21.6

Usage of LLINs	Yes	113	26.8
	No	309	73.2

Sleeping under LLINs	Daily	182	43.1
	Irregularly	24	5.7
	During malaria season	58	13.7
	Almost weakly	4	0.9
	Others specify^a^	5	1.2

IRS	Yes	107	25.4
	No	315	74.6

Holes *b*/*n* wall and roof of the household	Yes	186	44.1
	No	236	55.9

Availability of mosquito breeding site	Yes	294	69.7
	No	128	30.3

Proximity to the breeding sites	<1 km	60	14.2
	1-2 km	22	5.2
	>2 km	37	8.8

^a^other during treatment; LLINs = long-lasting insecticidal nets; IRS = residual indoor residual spraying.

**Table 4:** Bivariable and multivariable logistic regression analysis of malaria incidence and associated risk factors in Shewa Robit, Ethiopia (*n* = 422).

Variables	Malaria infection		COR (95% CI)			AOR (95% CI)		
	Negative *N* (%)	Positive *N* (%)						
*Age*								
<5	165 (89.7)	19 (10.3)	1			1		
5–14	110 (79.1)	29 (20.9)	2.3	1.22	4.29^*∗*^	2.31	1.15	4.65^*∗*^
15	67 (67.7)	32 (32.3)	4.15	2.20	7.82^*∗∗*^	4.05	1.95	8.42^*∗∗*^

*Sex*								
Male	157 (74.1)	55 (25.9)	2.59	1.54	4.35^*∗∗*^	3.24	1.75	5.97^*∗∗*^
Female	185(88.1)	25 (11.9)	1			1		

*Marital status*								
Married	124 (68.5)	57 (31.5)	4.37	2.56	7.42^*∗∗*^	4.97	2.67	9.28^*∗∗*^
Unmarried	218 (90.5)	23 (9.5)	1			1		

*Family size*								
<5	178 (88.1)	24 (11.9)	1			1		
≥5	164 (74.5)	56 (25.5)	2.53	1.50	4.27	2.20	1.2	4.06^*∗*^

*Usage of LLINs*								
Yes	95 (84.8)	17 (15.2)	1			1		
No	247 (79.9)	62 (20.1)	1.40	0.78	2.52	1.4	0.69	2.83

*IRS*								
Yes	94 (87.9)	13 (12.1)	1			1		
No	248 (78.7)	67 (21.3)	1.95	1.03	3.70	2.6	1.21	5.60^*∗*^

*Availability of a mosquito breeding site near to household*								
Yes	228 (77.6)	66 (22.4)	2.36	1.27	4.38	3.91	1.87	8.18^*∗∗*^
No	114 (89.1)	14 (10.9)	1			1		

*Hole b/n walls and roofs*								
Yes	139 (74.7)	47 (25.3)	2.08	1.27	3.41	2.1	1.13	3.61^*∗*^
No	203 (86.0)	33 (14.0)	1					

LLINs = long-lasting insecticidal nets, IRS = residual indoor residual spraying ^*∗*^ and ^*∗∗*^ indicate significance level at *p* < 0.05 and *p* < 0.001 respectively.

## Data Availability

All data generated or analyzed during this study were included in this published article; thus, no additional data were available.

## Abbreviations

ACT: Artemisinin combined therapy
AOR: Adjusted odds ratio
CDC: Centers for disease control and prevention
COR: Crude odds ratio
RDTs: Rapid diagnostic tests
PCR: Polymerase chain reaction
EMIS: Ethiopian national malaria indicator survey
IRS: Indoor residual spraying
ITNs: Insecticide treated nets
LLINs: Long-lasting insecticidal nets
WHO: World health organization.

## Supplementary Materials

Supplementary Materials Capillary blood collection and Malaria blood film preparation.
