# Supplementary material for: Prevalence and Associated Factors of Malaria Infection among Outpatients Visiting Shewa Robit Health Center, Northcentral Ethiopia
Source: J Trop Med. 2022 Mar 23;2022:1784012. doi: 10.1155/2022/1784012 (PMC8967545; doi:10.1155/2022/1784012)
Supplement: Supplementary Materials — Capillary blood collection and Malaria blood film preparation. [file 1784012.f1.pdf]

CAPILLARY BLOOD COLLECTION AND MALARIA BLOOD FILM PREPARATION

Purpose

This SOP provides instructions for drawing capillary blood from the finger (or earlobe and heel for infants) and preparing high-quality thick and thin malaria blood smears.

Principle

Capillary blood obtained by pricking the finger directly in adults or the earlobe or heel of the foot in infants. The blood is used immediately to form a thin and thick blood film, so it doesn't need an anticoagulant

Materials and supplies

1. Patient registration
2. Alcohol (70% ethanol)
3. Gemisa (3%)
4. Disposable sterile lancets
5. Absorbent cotton
6. Disposable gloves
7. Clean frosted end glass slides
8. Lead pencil
9. Slide drying tray
10. Biohazard container
11. Sharp container

Safety precautions

1. Wear protective gloves when handling or taking blood sample.
2. Cover any cuts or abrasions in your hands with adhesive dressing.
3. Always wash your hands with soap and water after handling the blood sample.
4. Take care not to accidentally prick yourself.
5. Never use a disposable lancet more than once.

Capillary blood collection procedure

1. Label the frosted end of the slide with patient ID numbers and dates.
2. Disinfect the finger or other sites thoroughly with an alcohol swab.
3. Let the alcohol dry.
4. Prick the site with a disposable sterile lancet, deep enough for blood to flow freely.
5. Wipe the first drop of the blood with dry cotton.
6. Apply gentle pressure to the finger / earlobe / heel to allow blood to flow
7. Discard the used lancets directly into the sharps disposal container.
8. From the pricked finger /earlobe/heel, collect blood directly in to the pre-labeled glass slides
9. Make thick and thin blood films on the same slide by touching the slide on the blood, place a small drop 2μl of blood in the middle portion of the slide and 1 bigger drop 6μl on the portion next to the frosted end.
10. Allow some space between the thick films to be made on the same slide.

Procedure for preparation of thin film

1. Quickly take a second clean and polished slide (spreader) and place it in front of the small drop of blood at an angle of 30-45°. Pull the slide back and hold until the blood has spread evenly over the age of the slide. Do not hesitate to apply and distributing the drop.
2. Quickly advance the slide in a single, smooth, continuous motion. Avoid hesitating or jerky movements when distributing blood.

Procedure for preparation of thick blood film

1. With one corner of the spreader slide, in a circular motion spread the blood out to make a circle with approximately 1cm in diameter, finishing off at the center.
2. The ideal thickness of the smear should allow for printed text to be readable when it is placed on it.
3. Discard the spreader in an appropriate slide container and do not re use it for another patient blood sample.
4. Allow both blood films to air dry in a horizontal position on slide tray. Slow drying prevents cracking. Avoid using a fan or blow dryer to dry these slides.

Cautions

A number of errors are common in making blood films this can affect the labeling, the staining or the examination.

A. Badly positioned blood films

Care must be taken to ensure that the blood smears are placed correctly on the slide. If this is not the case, it can be difficult to examine the thick film. in addition, some of the films may even be rubbed off during the dyeing or drying process.

B. Too much blood

After staining films made with too much blood:

- The background of the thick film will be too blue
- There will be too many white blood cells per thick field, and these could obscure or cover up any malaria parasites that are present.
- If the thin film is too thick, the red blood cells will be on top of each other and it will be impossible to examine them properly after fixation.

C. Too little blood

If too little blood is used to make the film:

- There will not be enough white cells in the thick film field and you will not examine enough blood in the standard examination.

D. Chipped edge of spreader slide

When the edge of slide chipped

- The thin film spreads unevenly, is streaky, and has many 'tails'
- The spread of the thick film may also be affected.

Quality control

Monitor the quality of the preparation of thick and thin films.

1. Follow proper collection procedure

2. Glass slides must be clean and free from greases.
3. Thick films and thin films must be prepared properly while drying protects blood film from dusts flies and insects
4. Do not dry express to direct sun light
5. Too thin a film may not have an adequate quantity of the blood for the detection of parasite.
6. Never add a pinch of EDTA powder directly to the sample tube.

Reference

1. WHO bench aids for diagnosis of malaria
2. RITM, parasitological manual of SOPs, august 2007
3. WHO Basic Malaria Microscopy, Learner Guide,2010 (second edition)

Declaration

We, undersigned agreed to strictly follow this SOP during capillary blood collection and preparation of blood films.

Azene Tesfaye
